# Urgences drépanocytaires au Service des Maladies du Sang du Centre National Hospitalier Universitaire-Hubert Koutoukou Maga de Cotonou, Benin

**DOI:** 10.11604/pamj.2018.30.192.15931

**Published:** 2018-07-03

**Authors:** Roger Dodo, Alban Zohoun, Tatiana Baglo, Josiane Mehou, Ludovic Anani

**Affiliations:** 1 Service des Maladies du Sang, Centre National Hospitalier Universitaire Hubert Koutoukou Maga de Cotonou, Benin; 2 Laboratoire d’Hématologie, Centre National Hospitalier Universitaire Hubert Koutoukou Maga, Cotonou, Benin; 3 Faculté des Sciences de la Santé de Cotonou, Université d’Abomey-Calavi, Bénin; 4 Hôpital d’Instruction des Armées-Centre Hospitalier Universitaire de Cotonou, Benin

**Keywords:** Urgences, drépanocytose, Bénin, Emergency treatments, sickle cell disease, Benin

## Abstract

**Introduction:**

L'évolution de la drépanocytose est marquée par la survenue de complications aigues dont certaines constituent de véritables urgences pouvant mettre en jeu le pronostic vital ou fonctionnel. Notre travail avait pour objectif d'étudier la fréquence et l'évolution des urgences drépanocytaires dans le Service des Maladies du Sang (SMAS) du Centre National Hospitalier Universitaire-Hubert Koutoukou Maga (CNHU-HKM) de Cotonou.

**Méthodes:**

Nous avons mené une étude rétrospective et descriptive de janvier 2014 à décembre 2015 et portant sur l'ensemble des patients hospitalisés pour une urgence drépanocytaire. Nous avons exclu les patients hospitalisés pour des complications drépanocytaires chroniques.

**Résultats:**

Deux cent quatre (204) urgences drépanocytaires pour un nombre total de 813 hospitalisations ont été enregistrés (prévalence de 25%). L'âge moyen des patients était de 24,2 ans. La tranche d'âge la plus représentée était celle de 20-30 ans (45,6%). Le sexe masculin prédominait à 60,8%. Les élèves/étudiants étaient les plus représentés à 55,9%. La douleur aigue était le motif principal d'hospitalisation à 63,7%. L'homozygotie SS était majoritairement représentée à 72,1%. Les complications vaso-occlusives ostéo-articulaires prédominaient à 70,1%. Les complications infectieuses documentées étaient dominées par le paludisme (27,5%). La décompensation anémique était notée à 30,4%. Sur le plan thérapeutique, l'hydratation a été utilisée à 85,3%. La durée moyenne de séjour était de 5,4 jours. L'évolution a été favorable dans 96,5% des cas. La létalité était de 2,5%.

**Conclusion:**

Les urgences drépanocytaires sont fréquentes. Elles nécessitent un diagnostic rapide et une prise en charge précoce et efficace. La formation continue du personnel médical à la prise en charge des urgences drépanocytaires s'avère nécessaire pour réduire leur mortalité.

## Abstract (en)

**Introduction:**

Evolution of sickle cell disease is marked by the occurrence of acute complications, some of which are real emergencies that can give rise to life-threatening or functional cosequences. This study aims to evaluate the frequency and the evolution of emergency treatment of sickle cell disease in the Blood Diseases Department at the Koutoukou Maga National Teaching Hospital, Cotonou.

**Methods:**

We conducted a retrospective and descriptive study of all patients hospitalized for emergency treatment of sickle cell disease from January 2014 to December 2015. We excluded patients hospitalized for chronic sickle cell disease complications.

**Results:**

Out of 813 hospitalizations, two hundred and four (204) emergency treatments of sickle cell disease were registered (prevalence 25%). The average age of our patients was 24.2 years. The most represented age group was 20-30 years (45.6%). Male sex predominated (60.8%). Pupils/students was the most represented group (55.9%). Acute pain was the primary reason for hospitalization to 63.7%. Normal homozygous individuals (SS) were mostly represented (72.1%). Osteoarticular vaso-occlusive complications predominated (70.1%). Documented infectious complications were dominated by malaria (27.5%). Decompensated anemia accounted for 30.4%. Therapeutic approach was based on hydration (85.3%). The average length of stay in hospital was 5.4 days. Outcome was favorable in 96,5% of cases. Mortality accounted for 2.5%.

**Conclusion:**

Emergency treatments of sickle cell disease are frequent. Early diagnosis as well as early and effective management are necessary. Ongoing training programs in emergency treatments of sickle cell disease are necessary to reduce mortality.

## Introduction

La drépanocytose est une maladie génétique autosomique récessive caractérisée par la présence d'une hémoglobine anormale, qui favorise la falciformation irréversible des hématies en situation d'hypoxie prolongée et aboutissant à des manifestations vaso-occlusives [1]. On regroupe sous le terme « syndrome drépanocytaire majeur », la forme homozygote SS et les formes hétérozygotes composites SC et S-β^+^thalassémie ou S-β^0^thalassémie qui ont une expression clinique voisine [2]. De nombreuses complications aiguës et chroniques, causes de morbidité font partie intégrante de cette affection. Les complications aiguës sont principalement représentées par les crises vaso-occlusives, l'anémie hémolytique chronique avec des épisodes d'aggravation aiguë et la susceptibilité aux infections bactériennes à germes encapsulés en raison de l'asplénie fonctionnelle [1-6]. Si de nombreuses études ont fait l'inventaire des complications de la drépanocytose, peu de données récentes sont disponibles sur les urgences drépanocytaires dans notre contexte. Face à ce constat, notre étude a pour but d'étudier la fréquence et l'évolution des urgences drépanocytaires en milieu hospitalier universitaire.

## Méthodes

Notre étude a été réalisée au Service des Maladies du Sang (SMAS) du Centre National Hospitalier Universitaire-Hubert Koutoukou Maga (CNHU-HKM) de Cotonou qui est l'hôpital de référence du Bénin. Clinique universitaire et unique service d'Hématologie Clinique du pays, le SMAS constitue le centre national de référence des pathologies hématologiques dont les hémoglobinopathies. Pour ces dernières, la prise en charge est globale et intègre les activités de prévention, de soins et de suivi. Les urgences drépanocytaires (médicale et chirurgicale) y sont directement admises et traitées avec une référence secondaire des cas chirurgicaux vers le service approprié. Il s'agit d'une étude rétrospective descriptive sur une période de 2 ans (Janvier 2014-Décembre 2015) et portant sur un recrutement exhaustif de tous les patients admis pour une urgence drépanocytaire et hospitalisés dans le service. Les critères d'inclusion étaient représentés par l'existence des urgences suivantes : les anémies aigues ; les infections graves (sepsis, ostéomyélites, méningites, pneumopathies,…) auxquels on peut rattacher le paludisme comme un facteur favorisant ; la crise vaso-occlusive osseuse (CVO); le syndrome thoracique aigu; le priapisme; les accidents vasculaires aigus (accident vasculaire cérébral, atteinte vestibulaire ou cochléaire, nécrose papillaire, infarctus viscéral, hémorragie ophtalmologique, thrombose veineuse) et les urgences aigues secondaires aux complications chroniques. Les critères de non inclusion étaient représentés par les hospitalisations pour complications drépanocytaires chroniques et ne relevant pas de l'urgence (ulcères cutanés, rétinopathie proliférante, néphropathie drépanocytaire, atteinte cardiaque). La grossesse représente une situation particulière où les patientes sont hospitalisées en obstétrique et font l'objet d'une prise en charge pluridisciplinaire. Notre échantillon était composé en majorité de drépanocytaires connus et faisant partie de la cohorte suivie dans notre service. Les moyens diagnostiques utilisés étaient cliniques (antécédents, examen physique), biologiques (hémogramme, bilans d'hémostase, électrophorèse quantitative de l'hémoglobine, bilans biochimique et microbiologique) et radiologiques (radiographie standard, échographie et scanner). Les avis spécialisés notamment ophtalmologique, oto-rhino-laryngologique, hépato-gastrologique, cardiologique et néphrologique étaient sollicités si besoin. L'évolution était jugée favorable devant l'amélioration de l'état général et la sortie du patient et défavorable en cas de décès. Les variables étudiées étaient les caractéristiques socio-démographiques (âge, sexe, profession); le motif de consultation; le type d'hémoglobinopathie (homozygotie SS, hétérozygotie composite SC ou S-β-thalassémie); le diagnostic retenu; le traitement reçu ; l'évolution et la durée d'hospitalisation. La collecte des données a été faite à partir des registres d'hospitalisation et des dossiers des patients à l'aide d'une fiche pré-testée. Les données ont été codées, enregistrées et analysées à partir des logiciels Excel et SPSS 20.0. Nous avons ressorti les pourcentages pour les variables quantitatives. Pour garantir la confidentialité des données, les fiches ont été conçues de façon à respecter l'anonymat des patients.

## Résultats

**Caractéristiques socio-démographiques:** Nous avons recensé 204 hospitalisations non redondantes pour urgences drépanocytaires pour un nombre total de 813 hospitalisations, soit une prévalence hospitalière de 25%. L'âge moyen des patients était de 24,2 ans avec des extrêmes allant de 5 à 59 ans. La tranche d'âge la plus représentée était celle de 20-30 ans (45,6%). Une prédominance masculine a été retrouvée à 60,8% avec un sex-ratio (H/F) de 1,55. Les élèves/étudiants étaient les plus représentés à 55,9% suivis des ouvriers à 21,6%. Le Tableau 1 résume les caractéristiques socio-démographiques de notre échantillon.

**Tableau 1:** Récapitulatif des caractéristiques sociodémographiques

Variables	N	%
**Tranche d’âge (années)**		
[0- 10[	10	4,9
[10- 20[	53	26
[20- 30[	93	45,6
[30- 40[	29	14,2
³ 40	19	9,3
**Sexe**		
Masculin	124	60,8
Féminin	80	39,2
**Profession**		
Ouvrier	44	21,6
Elève/étudiant	114	55,9
Revendeuse	13	6,4
Ménagère	5	2,4
Fonctionnaire	20	9.8
Non précisée	8	3.9
**Résidence**		
Cotonou	139	68,1
Hors Cotonou	45	22,1
Non précisée	20	9,8

**Motif d'hospitalisation et type d'hémoglobinopathie:** Le motif d'hospitalisation le plus fréquent était la douleur aigue (63,7%). Les localisations hyperalgiques étaient ostéo-articulaire (78,5%), abdominale (2,5%), thoracique (3,2%), pénienne (4,4%) et multifocale (11,4%). La cohorte de syndromes drépanocytaires majeurs dans notre série était composée de 147 drépanocytaires homozygote SS (72,1%) et 57 hétérozygotes composites SC (27,9%) (Tableau 2).

**Tableau 2:** Récapitulatif des caractéristiques cliniques

Variables	N	%
**Motif de consultation**		
Douleurs aigues	130	63,7
Fièvre	11	5,4
Pâleur	4	2
Asthénie	15	7,3
Douleur fébrile	24	11,8
Autres	20	9,8
**Localisation de la douleur**		
Ostéo-articulaire	124	78,5
Abdominale	4	2,5
Thoracique	5	3,2
Pénienne	7	4,4
Multifocale	18	11,4
**Type d’hémoglobinopathie**		
SS	147	72,1
SC	57	27,9

**Urgences drépanocytaires:** Concernant les urgences drépanocytaires, les localisations des CVO se répartissaient comme suit : ostéo-articulaire (70,1%), abdominale (12,7%), thoracique (4,9%), pénienne (4,9%) et cérébrale (1%). Les complications infectieuses étaient représentées par : les infections bactériennes sans foyer localisé (27,9%), les pneumopathies (18,6%), les ostéomyélites (3,9%) et la pyélonéphrite (2,9%). Le paludisme, facteur favorisant des CVO était retrouvé à une fréquence de 27,5%. La décompensation anémique était retrouvée à 30,4%. Les urgences drépanocytaires sont résumées dans le Tableau 3.

**Tableau 3:** Répartition des urgences drépanocytaires

Urgences drépanocytaires	N	%
**Crises vaso-occlusives**		
Ostéo-articulaire	143	70,1
Abdominale	26	12,7
Priapisme	10	4,9
Syndrome thoracique aigu	10	4,9
AVC	2	1
**Décompensation anémique**		
anémie décompensée	62	30,2
**Complications infectieuses**		
Pneumonie	38	18,6
Infection bactérienne sans foyer localisé	57	27,9
Ostéomyélite	8	3,8
Pyélonéphrite	6	3,9

**Thérapeutiques instituées:** Les traitements médicamenteux étaient représentés par : les antalgiques de palier I (73,5%), palier II (59,8%) et palier III (23%) ; les anti-inflammatoires non stéroïdiens (40,2%) ; les antibiotiques (45,6%) et les anti-malariques à 27,5%. Une hydratation était presque toujours associée dans 85,3% des cas. La ponction caverneuse en cas de priapisme a été réalisée dans 2% des cas. La transfusion de culot globulaire a été réalisée dans 29,4% des cas et une transfusion d'échange partielle dans 2% des cas. L'oxygénothérapie était systématique pour toute situation de saturation en oxygène (SaO2) < 96%.

**Evolution et durée d'hospitalisation:** L'évolution était favorable dans 96,5% des cas. La durée moyenne d'hospitalisation était de 5,4 jours avec des extrêmes allant de 1 à 20 jours. La létalité était de 2,5% (Tableau 4).

**Tableau 4:** Récapitulatif des caractéristiques évolutives

Evolution	N	%
Favorable	197	96,5
Décès	5	2,5
Sortie contre avis médical	2	1
**Causes de décès**		
Décompensation anémique	2	40
Syndrome thoracique aigu	3	60

## Discussion

Le SMAS, unique Service d'Hématologie Clinique au Bénin, constitue le centre de référence de prise en charge des hémopathies en général et des hémoglobinopathies en particulier. Aussi, notre échantillon d'étude peut être considéré comme représentatif du fait que le recrutement a été fait à Cotonou, cosmopolite et principale ville du pays. Le syndrome drépanocytaire majeur se traduit cliniquement par des symptomatologies nombreuses et variées pouvant dans certains cas mettre en jeu le pronostic vital. Ces complications, pour l'essentiel aigues, constituent des urgences et nécessitent une prise en charge spécifique et bien codifiée. Les urgences drépanocytaires représentaient un quart (25%) des hospitalisations enregistrées dans le service. Ce pourcentage est inférieur à celui de 78,42% retrouvé par Latoundji et al. en 1990 [7]. Cette différence est liée au fait que les auteurs s'étaient intéressés à toutes les morbidités de la drépanocytose (complications aigues et chroniques) contrairement à notre étude qui ne s'est intéressée qu'aux complications aigues. L'âge moyen des patients dans notre série était de 24,2 ans. Perignon et al [8] ont rapporté respectivement des résultats similaires avec un âge moyen de 25 ans. Le sexe masculin était prédominant à 60,8% avec un sexe ratio H/F de 1,55, identique au constat rapporté par Mbika et al dans leur étude [9]. La drépanocytose homozygote SS prédominait dans notre étude (72,1%). Gbadoé et al [10] ont rapporté une prévalence similaire de cette forme à 68,2%. Le motif d'hospitalisation le plus fréquent était la douleur aigue, ce qui est conforme aux données de la littérature [11-14]. La localisation de ces douleurs aigues était à prédominance ostéo-articulaire avec une fréquence de 78,5%. Elle est supérieure à celle rapportée par Gbadoé et al [10] et Mbika et al [9] avec une fréquence respective de 47,6% et 55%. Ce sont des douleurs essentiellement osseuses, plus rarement articulaires, dont l'intensité est parfois comparée à celle d'une fracture osseuse [14]. L'intensité, la répétition, le caractère angoissant et imprévisible des crises douloureuses entraînent un retentissement fonctionnel majeur. Le retentissement psychologique est d'autant plus fort que la douleur apparaît sous-estimée par les soignants [15]. Le syndrome thoracique aigu est la première cause de mortalité chez les drépanocytaires et constitue le deuxième motif d'hospitalisation après la crise vaso-occlusive simple [16-17]. C'est la complication sévère la plus fréquente au cours des CVO et elle est source d'une mortalité et d'une morbidité importante [18]. Il est retrouvé à une fréquence de 4,9% dans notre série, beaucoup moindre que celui de 17,8% rapporté par Bartolucci et al [18].

La fréquence du priapisme était de 4,9%. Cette complication aigue représente une urgence absolue car le pronostic fonctionnel est mis en jeu par un risque de fibrose des corps caverneux pouvant entraîner une impuissance en cas de priapisme prolongé [1]. Les interactions entre drépanocytose et paludisme restent obscures et la croyance selon laquelle les sujets drépanocytaires seraient protégés reste répandue [8]. A l'opposé de ces affirmations, le paludisme documenté par une goutte épaisse positive, facteur favorisant des CVO était retrouvée à 27,5%. Ce constat est corrobé par Mbika et al [9] qui le rapportent à une fréquence de 18%. Les pneumopathies représentent 18% des complications infectieuses. Cette fréquence est identique à celle 17% retrouvée par Mbika et al [9]. La décompensation anémique représentait 30,4% des urgences drépanocytaires. Mbika et al [9] dans leur série ont rapporté une fréquence de 37%. Étant donné l'hémolyse permanente, la chute de l'hémoglobine est brutale si l'érythropoïèse devient insuffisante [14]. L'hydratation demeure une mesure générale incontournable dans le traitement des crises douloureuses vaso-occlusives. Elle doit apporter au maximum 3 litres/jour de solutés ou d'eau de boisson par jour si les fonctions cardiaque et rénale sont normales [19]. L'hydratation était le moyen thérapeutique le plus utilisé dans 85,3% des cas dans notre étude. La prédominance des complications vaso-occlusives explique cette attitude thérapeutique. Les autres moyens thérapeutiques étaient institués en fonction du tableau clinique. L'évolution sous traitement était favorable dans 96,5% avec régression puis sédation des manifestations aiguës. Le programme de suivi des drépanocytaires dans le service, le recours rapide aux soins et la disponibilité d'un personnel spécialisé et qualifié peuvent expliquer ce taux de succès qui est à souligner. Cinq décès, tous chez des drépanocytaires SS, ont été retrouvés dont deux cas de décompensation anémique brutale (décès en rapport avec une pénurie de sang) et trois cas de syndrome thoracique aigu. Latoundji S et al [7] rapportaient un taux de mortalité globale de 10,61% pour la drépanocytose SS et que l'anémie était l'un des facteurs importants associés à la survenue de décès. L'amélioration continue de la disponibilité en produits sanguins (promotion du don, formation du personnel et utilisation rationnelle du sang) associée à une surveillance rapprochée et la création d'unité de réanimation hématologique efficace permettront de réduire la mortalité liée à ces deux étiologies de morbidité.

## Conclusion

Il ressort de cette étude menée en milieu hospitalier que les complications aiguës de la drépanocytose sont précoces et fréquentes. Elles surviennent dans la majorité des cas chez un sujet adulte jeune et de sexe masculin. Les principales urgences drépanocytaires étaient dominées par les crises vaso-occlusives ostéo-articulaires, les infections bactériennes et l'anémie aiguë. L'anémie aiguë et le syndrome thoracique aigu constituent les deux causes de mortalité. Le programme de suivi des patients, l'amélioration du plateau technique, la codification et la rapidité de la prise en charge en milieu spécialisé modifient le devenir des patients en réduisant la morbi-mortalité.

### Etat des connaissances actuelles sur le sujet

- La drépanocytose est un problème de santé publique en Afrique subsaharienne de par sa morbi-mortalité. Au Bénin, la prévalence du trait drépanocytaire S est estimée à 20% et celle de l'hémoglobine C à 10%. De même, on estime à environ 5% le pourcentage de la population porteur de l'homozygotie SS et la double hétérozygotie SC;
- Les complications aiguës souvent s'intrinquent et mettent en jeu le pronostic vital quand la prise en charge n'est pas précoce et spécialisée. Les principales complications aiguës sont représentées par les crises vaso-occlusives, l'anémie aiguë, les infections surtout bactériennes, le syndrome thoracique aigu, le priapisme et les accidents vasculaires cérébraux.

### Contribution de notre étude à la connaissance

- Cette étude fournit des données sur les caractéristiques épidémiologiques, cliniques, thérapeutiques et évolutives des urgences drépanocytaires au Bénin;
- L'anémie aiguë et le syndrome thoracique aigu constituent les deux causes de mortalité des urgences drépanocytaires, soulignant les efforts et moyens à mettre en œuvre pour améliorer la prise en charge des patients.

## Conflits d’intérêts

Les auteurs ne déclarent aucun conflit d'intérêts.

## References

[1] Habibi A, Arlet JB, Stankovic K, Gellen-Dautremera J (2015). Recommandations françaises de prise en charge de la drépanocytose de l'adulte: actualisation 2015. Rev Med Interne..

[2] Godeau B (2000). La drépanocytose chez l'adulte: quelles urgences pour l'interniste. Rev Med Interne..

[3] Ephraim RK, Osakunor DN, Cudjoe O, Oduro EA (2015). Chronic kidney disease is common in sickle cell disease: a cross-sectional study in the Tema Metropolis, Ghana. BMC Nephrology.

[4] Colella MP V de, Paula U, Machado-Neto JA, Conran N (2015). Elevated hypercoagulability markers in hemoglobin SC disease. Haematologica.

[5] Napon C, Kaboré A, Ouédraogo M, Dravé A (2012). Accidents vasculaires cérébraux et hémoglobinopathies au Burkina Faso. Med Sante Trop..

[6] Ngo Sack F, Seck M, Faye B, Diop S (2016). Morbidité et aspects évolutifs de la drépanocytose SC: une étude de 129 patients au Service d'Hématologie Clinique de Dakar. Health Sci Dis.

[7] Latoundji S, Anani L, Ablet E, Zohoun I (1991). Morbidité et mortalité drépanocytaire au Bénin. Med Afr Noire..

[8] Perignon A, Botterel F, Farrugia C, Foulet F (2008). Paludisme et drépanocytose homozygote. Med Mal Infect..

[9] Mbika Cardorelle A, Okoko A, Mouko A (2010). Les crises vaso-occlusives de l'enfant drépanocytaire à Brazzaville. Arch Pediatr.

[10] Gbadoé AD, Atakouma DY, Akoli A, Assimadi JK (1999). Traitement à domicile de la crise vaso-occlusive drépanocytaire au Togo. Arch Pediatr.

[11] Brousseau DC, Owens PL, Mosso AL, Panepinto JA (2010). Acute care utilization and rehospitalizations for sickle cell disease. JAMA.

[12] Lanzkron S, Carroll CP, Haywood CJr (2010). The burden of emergency department use for sickle-cell disease: an analysis of the national emergency department sample database. Am J Hematol..

[13] Platt OS, Thorington BD, Brambilla DJ, Milner PF (1991). Pain in sickle cell disease: rates and risk factors. N Engl J Med..

[14] Lionnet F, Arlet JB, Bartolucci P, Habibi A (2009). Recommandations pratiques de prise en charge de la drépanocytose de l'adulte. Rev Med interne..

[15] Anie KA (2005). Psychological complications in sickle cell disease. Br J Haematol..

[16] Platt OS, Brambilla DJ, Rosse WF, Milner PF (1994). Mortality in sickle cell disease: life expectancy and risk factors for early death. N Engl J Med..

[17] Vichinsky EP, Neumayr LD, Earles AN, Williams R (2000). Causes and outcomes of the acute chest syndrome in sickle cell disease: national Acute Chest Syndrome Study Group. N Engl J Med..

[18] Bartolucci P, Habibi A, Khellaf M, Roudo-Thoraval F (2014). Score prédictif de survenue d'un syndrome thoracique durant une crise vaso-occlusive drépanocytaire dès l'arrivée aux urgences. Rev Med Interne..

[19] Ferster A, Kentos A, Bradstreet C, Vertongen F (2005). Traitement de la crise douloureuse vaso-occlusive. Jeur.

